# Phase Separation Mediated Sub-Nuclear Compartmentalization of Androgen Receptors

**DOI:** 10.3390/cells13201693

**Published:** 2024-10-13

**Authors:** Selçuk Yavuz, Tsion E. Abraham, Adriaan B. Houtsmuller, Martin E. van Royen

**Affiliations:** 1 Department of Pathology, Erasmus University Medical Center, Doctor Molewaterplein 40, 3015 GD Rotterdam, The Netherlands; s.yavuz@erasmusmc.nl (S.Y.); m.vanroyen@erasmusmc.nl (M.E.v.R.); 2 Erasmus Optical Imaging Center, Erasmus University Medical Center, Doctor Molewaterplein 40, 3015 GD Rotterdam, The Netherlands; t.abraham@erasmusmc.nl (T.E.A.)

**Keywords:** nuclear receptors, subnuclear compartmentalization, gene transcription regulation, liquid–liquid phase separation, prostatic neoplasms

## Abstract

The androgen receptor (AR), a member of the nuclear steroid hormone receptor family of transcription factors, plays a crucial role not only in the development of the male phenotype but also in the development and growth of prostate cancer. While AR structure and AR interactions with coregulators and chromatin have been studied in detail, improving our understanding of AR function in gene transcription regulation, the spatio-temporal organization and the role of microscopically discernible AR foci in the nucleus are still underexplored. This review delves into the molecular mechanisms underlying AR foci formation, focusing on liquid–liquid phase separation and its role in spatially organizing ARs and their binding partners within the nucleus at transcription sites, as well as the influence of 3D-genome organization on AR-mediated gene transcription.

## 1. Introduction

Androgen receptors (ARs) are steroid hormone receptors (SHR) and are important for the development and maintenance of the male reproductive system by regulating growth and differentiation [1]. Furthermore, ARs play a predominant role during the development and growth of prostate cancer (PCa), which has the second highest incidence among all cancers in males [2]. Androgens such as testosterone and its more potent metabolite, 5α-dihydrotestosterone (DHT), bind to the AR and induce its translocation into the nucleus. ARs then bind to DNA at androgen-responsive element (ARE) sequences within AR-binding sites (ARBS) as monomers and dimers to orchestrate gene transcription [3,4]. One of the characteristics of the AR, similar to many other transcription factors (TF), is its capacity to form intranuclear accumulations referred to as biomolecular foci, speckles, or condensates [5,6,7]. AR foci are reported to correlate with the gene transcription activity of ARs, which has led to the hypothesis that AR-regulated gene transcription might be facilitated inside these structures [8,9,10,11]. Here, we review the current view on the functional significance of AR foci and the molecular factors influencing the formation of these structures and propose a framework for understanding this process through an AR phase separation model in the context of gene regulation and the 3D genome.

## 2. Androgen Receptor-Mediated Gene Transcription

**Figure 1:**
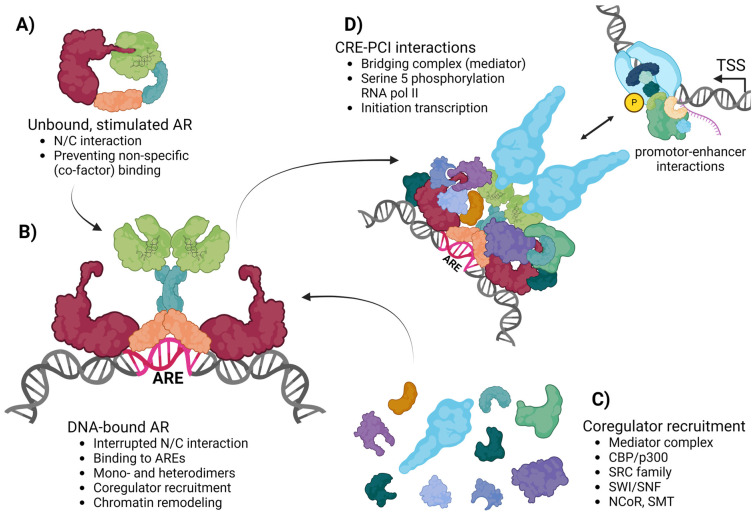
AR and its coregulators: schematic representation of the AR’s mode-of-action starting from hormonal stimulation (**A**), followed by DNA-binding at AR-binding sites (ARBS) containing androgen-responsive elements (AREs) at CIS-regulatory elements (CISs) as either monomer (not shown) or homodimer (**B**). Subsequently, AR interacts with various coregulators (**C**) to eventually initiate gene transcription by phosphorylating serine 5 of RNA polymerase II of the preinitiation complex (PIC) via the mediator complex (**D**).

Advancements in the fields of gene transcription and genome organization have significantly enhanced our understanding of how transcription factors, such as ARs, execute their biological functions. After hormonal activation and translocation into the nucleus, ARs bind to thousands of ARBS at mainly cis-regulatory elements (CRE) located in intergenic regions, followed rapidly by foci formation [12,13,14]. ARs binding to these sites depends on several local features of the chromatin, such as the state of chromatin compaction, ARE motif sequences, and local cofactor interactions, which contribute to the stabilization of DNA-bound ARs. Moreover, ARs are also capable of inducing local chromatin remodeling via coregulators, which, in turn, allow the physical interaction between ARs and their interactome at CREs. This interaction involves proteins from the transcription preinitiation complex (PIC) at promoter regions, which are essential for regulating gene transcription [15,16,17,18]. The modification of chromatin during transcriptional processes involving ARs requires several steps. In the initial phase, pioneer factors such as FOXA1 and GATA4 are required to enable the direct binding of ARs within compacted chromatin regions [19]. Subsequently, a diverse series of histone modifications are employed to further loosen the chromatin structure, including histone acetyltransferases (HATs) and histone demethylases (HDMT) [15,20]. Among the coactivators frequently associated with ARs, SRC1 and SRC3 exhibit moderate HAT activity. Importantly, they can also form interactions with other cofactors possessing stronger HAT activity, such as P300 and CBP [21]. These coactivators also have the ability to modify other protein complexes and facilitate the binding of SWI/SNF chromatin remodelers to ARs and their interactome, which, in turn, enhance the accessibility of chromatin further to allow the interaction between ARs located at CREs and RNA polymerase II at the PIC via the mediator bridging complex [22,23,24,25]. As a result, MED1, a member of the mediator complex, induces gene transcription by phosphorylating serine 5 of RNA polymerase II (Figure 1).

The development of advanced microscopy technologies to measure the biophysical properties of proteins in living cells, such as fluorescence recovery after photobleaching (FRAP), fluorescence correlation microscopy (FCS), and single-particle tracking (SPT), has enabled researchers to link protein dynamics to their biological roles [26,27,28,29]. These techniques have revealed that biological processes like gene transcription and DNA repair are inherently stochastic and dynamic. For example, nuclear proteins like SHRs show fast diffusion, interrupted by periods of DNA-binding with immobilization times lasting from several seconds to a few minutes [26,29,30]. This highly dynamic behavior of SHRs is thought to be crucial for the finely tuned regulation of gene transcription of target genes [4,26,30,31]. Despite their high mobility, the intranuclear foci formed by SHRs are spatio-temporally conserved [12,32,33]. Although the mechanism of AR-mediated gene transcription is well-documented at the molecular level, gaps remain in our comprehension of the mechanistic link between the microscopically discernible AR foci within the nucleus and the dynamic AR-mediated gene transcriptional activity.

## 3. Spatial Organization and Androgen Receptor Function

**Figure 2:**
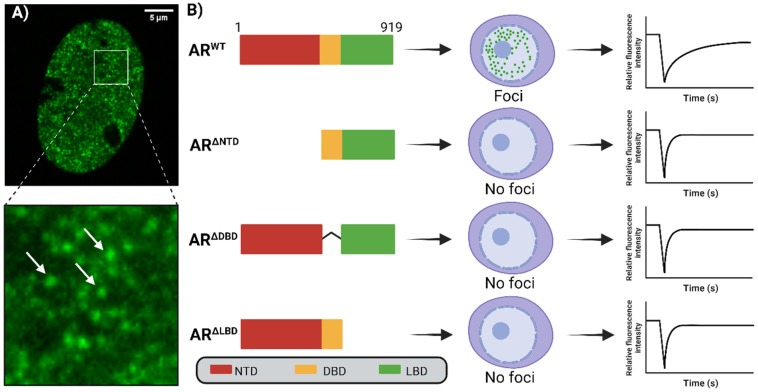
Relationship between AR structural status and LLPS: (**A**) Confocal image of a cell expressing testosterone-stimulated EGFP-AR. Arrows indicate the location of some well-defined AR foci as example. (**B**) AR full-length wild type consists of an N-terminal domain (NTD), DNA-binding domain (DBD) and Ligand-binding domain (LBD). Upon stimulation by testosterone and other (synthetic) derivates, the AR wild type forms intranuclear foci whereas AR truncated mutants lacking either the NTD, DBD or the LBD (e.g., ARv7) are not able to form foci. Biophysical measurements using fluorescence recovery after photobleaching (FRAP) revealed that AR WT diffuses the slowest over time whereas the AR truncated mutants lacking the NTD, DBD or the LBD are significantly faster diffusing.

ARs form protein condensates consisting of AR molecules near ARBS, resulting in a nuclear-speckled pattern similar to nuclear speckles associated with mRNA production and splicing (Figure 2A) [12,34]. The presence of an AR dimer at a single ARBS is insufficient to resemble the microscopically discernable foci observed by microscopy and requires tens to several hundreds of fluorescently labeled ARs for detection. Therefore, nucleated ARs from several proximal ARBS may be spatially positioned in close proximity, which contributes to the formation of foci. Like nuclear speckles, AR foci have been shown to correlate microscopically with specific DNA regions containing ARE sequences in living cells upon stimulation by androgens [12]. These foci have also been shown to coincide primarily with euchromatin while partially colocalizing with sites enriched with active histone marks, such as H3K27Ac [12]. Furthermore, they also overlap with activated serine 5-phosphorylated RNA polymerase II and nascent RNA, indicating that these foci represent sites of active gene transcription [4,12,25]. However, a significant population of AR foci does not colocalize with these active transcription sites. It is hypothesized that these AR foci may be involved in downregulating gene transcription by recruiting corepressors, such as NCoR and SMRT, instead of coactivators, which, in turn, recruit histone deacetylases (HDACs) to induce chromatin compaction [4,12,35,36,37,38].

Like all members of the SHR family, ARs comprise three main subdomains: the N-terminal domain (NTD), the DNA-binding domain (DBD), and the ligand-binding domain (LBD) (Figure 2B) [39,40]. Deleting either the NTD, which contains the primary transactivation function (AF1) region, or the DBD, with its DNA-binding function, significantly reduces the transactivation capacity of the AR. However, AR variants lacking the LBD, such as the clinically relevant ARv7, maintain strong transcriptional activity (shown by plasmid luciferase assays and differential mRNA expression of AR-regulated genes) through the NTD [41,42,43]. Interestingly, cells expressing fluorescent-labeled ARs lacking one of the functional domains—NTD, DBD, or the LBD—show a complete loss of foci formation, indicating that all these domains are necessary for AR foci generation [12,42,43,44] (Figure 2B). Moreover, analysis of their dynamic behavior, for instance, using FRAP microscopy, showed a direct relationship between the capability of wild-type and mutant ARs to stably bind DNA and the formation of nuclear foci (Figure 2B) [31,45,46]. The obvious functional importance of the DBD and DNA-binding in foci formation was shown by single missense mutations in the residues in the DBD that directly interact with DNA, such as R585K, preventing ARs from forming foci by disrupting their interaction with DNA. The use of antiandrogens (e.g., enzalutamide, hydroxyflutamide, or bicalutamide) also results in a complete absence of AR foci, accompanied by transcriptional inactivity of wild-type AR, similar to AR DBD mutants, indicating that antiandrogens prevent stable AR-DNA-binding [9]. Where the DNA-binding deficient AR mutant is transcriptionally inactive, LBD-truncated ARs remain transcriptional active despite their low level of stable DNA-binding [42]. The apparent dispensability of the AR-LBD for transcriptional activity is also reflected by the clinically relevant truncated AR variants (e.g., ARv7) and can be explained by the relatively weak transactivation function of the LBD compared to the NTD, a specific property of AR. In contrast, SHRs in which the transactivation capacity of the receptor lies more towards the LBD, such as ER, lose their transactivation capacity as well as foci formation upon LBD removal [47,48]. The predominant role of the AR’s NTD is further emphasized by the reduced foci formation and inhibited transcriptional activity in agonist-activated ARs treated with small molecule compounds (e.g., EPI-001, its newer derivate EPI-7386 and ET516). These compounds intercalate with the transactivation unit 5 (TAU5), responsible for cofactor interactions (e.g., SRC1, FOXO1), located at the C-terminal region of the NTD [8,49,50]. Therefore, it can be hypothesized that these inhibitors prevent the binding of cofactors to the NTD, which, in turn, reduces stable DNA-binding and foci formation [51]. However, since direct NTD-DBD interactions were also reported, it cannot be excluded that they indirectly affect—independently from cofactors—the capacity of the DBD to bind DNA [49]. Together, this indicates the importance of the AR’s stable DNA binding in foci formation and their role in transcriptional activity. 

Given the critical role of coregulators in stabilizing SHR transcriptional complexes on the DNA, numerous studies have focused on understanding their function in the context of foci formation. Coregulators often form protein condensates with SHR, while the expression of certain coregulators can inhibit foci formation. For example, a repressive CBP protein variant hinders AR foci formation [52]. Among the known SHR interactors, the SRC proteins are often described in the context of SHR foci, as it has been shown that corepressors compete with SRC coactivators, such as SRC1, for recruitment to AR foci to regulate their formation [53]. Additionally, dephosphorylation of SRC1 significantly reduces PR foci presence [54], whereas mutations in SRC2-binding sequences on GR resulted in reduced GR foci [55], underscoring the importance of coregulator-SHR interactions in foci formation, potentially because they increase the stability of SHRs on the DNA. In addition, antiandrogens targeting the AR ligand-binding pocket are thought to induce the binding of corepressors at ARBS [37], influencing foci composition by recruiting HDACs and acting on histones and cofactors [56,57]. However, this is contradicted by the lack of stable DNA-binding and AR foci formation by ARs treated with antagonists, similar to AR DBD mutants [9]. Nevertheless, it cannot be excluded that stable DNA-binding is not required for transcriptional repression, similar to LBD-truncated mutants who are not stably DNA-bound but still able to regulate gene transcription via short DNA interactions. 

Although the cofactor-binding groove in the ligand-activated LBD is occupied by the FQNLF motif in the NTD (N/C interaction) in an AR monomer or dimer conformation, a combination of fluorescence resonance energy transfer (FRET) and FRAP microscopy showed that the NTD interaction with the cofactor-binding groove in the LBD is lost in the stably DNA-bound AR (Figure 1) [4,42]. Functional analysis of the cofactor-binding groove with an ARA54 cofactor FXXLF peptide in living cells indeed confirmed that the loss of the competitive AR N/C interaction in DNA-bound ARs allows interactions with cofactors via the cofactor-binding groove [4]. This is in apparent contradiction with a cryo-electron microscopy-based structural analysis of isolated DNA-bound AR dimers, which showed that the LBDs and DBDs are at the core of the DNA-bound AR complex, likely facilitated by direct DBD-DBD and LBD-LBD dimerization, with the two NTDs wrapping around the LBDs to contact each other, suggesting that AR-cofactor interactions occur mainly via the NTD [58]. However, EM images are static, and it may be that the more open protein complex confirmations are missed in this analysis. Protein interaction studies revealed that cofactors (e.g., SRC coactivators) can bind to both the NTD and the LBD, providing evidence for the structural and functional role of both domains in AR-cofactor interactions [58,59,60,61]. However, only the loss of NTD-cofactor interactions, and not the LBD-cofactor interactions, strongly affect transcriptional activity [59]. For example, I182A/L183A mutations impair the AR’s transcriptional activity (but not the intrinsic transcriptional activity of the NTD) as a consequence of disrupted NTD-LBD interactions, confirming that the NTD and LBD work cooperatively to efficiently facilitate coactivator recruitment.

## 4. Androgen Receptor Mode of Action in the Context of the 3D Genome 

Technological advancements in the field of high-throughput sequencing technologies have deepened our knowledge of how SHRs act in a 3D genome context. Chromatin immunoprecipitation in combination with sequencing (ChIP-Seq) unveiled a genome-wide pattern of AR-DNA interactions, while analysis of transcript levels across the entire genome, employing methodologies such as microarrays or RNA sequencing (RNA-Seq), confirmed that ARs exert a regulatory influence over the expression of genes at these sites [62,63,64]. The majority of identified binding sites are situated mainly within intergenic regions, often distant from gene coding sequences and promoters, frequently corresponding instead to CIS-regulatory elements (CREs), such as enhancers [65,66,67,68]. 

**Figure 3:**
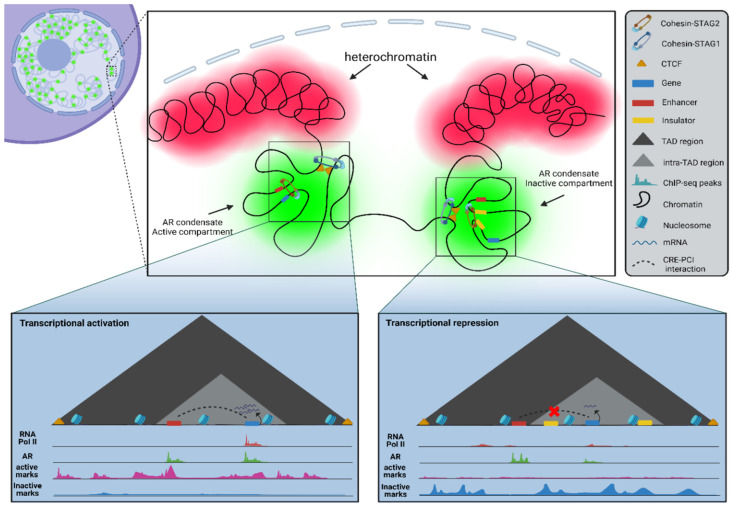
Three-dimensional chromatin regulation in context of AR biology. ARs are located at transcriptionally active and inactive regions, which are topologically organized in well-separated chromatin domain (TADs) by the cohesin complex, mainly located at euchromatic regions. Active and inactive TADs are characterized by presence of transcriptionally initiating or repressing epigenetic marks. ARs play role during Intra-TAD interactions, such as promotor-enhancer or promotor-insulator interactions, by potentially recruiting mainly cohesin-STAG2 complexes at enhancers. Outcome of this regulation depends on the type of CIS-regulatory elements (enhancer or insulator) present in the (intra-)TAD region, alongside the epigenetic status and chromatin compaction of the locus.

SHRs mostly regulate gene transcription via CRE sites by forming DNA loops to bring the enhancer or insulator CRE site physically closer to the PIC at promotor regions. The formation of loops depends on the overall genome organization. Chromosome paint analysis has shown that interphase chromosomes are largely segregated within the nucleus with minimal surface intermingling [69,70]. Chromosome conformation capturing techniques (3C) have confirmed the existence of these interactions, mostly characterized as intrachromosomal interactions, as expected based on a stochastically driven closer proximity (on average) of loci within a chromosome compared to loci in two different randomly positioned chromosomes within nuclei [71,72,73,74]. High-resolution contact maps have shown that these highly conserved intrachromosomal interactions are separated by sharp genomic boundaries, referred to as topologically associated domains (TADs), that are held together by chromosome structuring complexes such as cohesin [75,76,77]. Within TADs, various epigenetic marks promote or suppress gene transcription [78] by facilitating or blocking spatial interactions between CREs and promoters, aided by cohesin complexes during loop extrusion processes (Figure 3) [79]. However, local chromatin decompaction and activating histone marks, mainly H3K27Ac and H3K4me3, do not always result in AR-induced transcription activation. Downregulated AR target genes and their corresponding CREs also often show these modifications along accessible chromatin, suggesting an additional regulatory process. Interestingly, these downregulated genes, despite their activation marks, displayed decreased chromatin looping frequency with their CREs, highlighting chromatin looping as crucial for AR transcription activation alongside epigenetic modifications and the state of chromatin compaction [80]. In PCa, several TAD rearrangements were reported, causing upregulation of oncogenes (e.g., AR and FOXA1) and downregulation of tumor suppressor genes (e.g., TP53) by affecting the CRE-PIC interactions [81,82,83,84]. These enhancer-promoter loops are often enriched in PCa for ARs and its pioneer factor FOXA1 to co-regulate gene transcription within TAD boundaries defined by CTCF and cohesin [84]. Loop extrusion’s role in AR biology was further supported by reports describing elevated enhancer-promoter interactions upon AR hormonal stimulation, occurring mainly via pre-established promoter-enhancer interactions rather than rewiring new local chromatin contact loops initiated by ARs [80,85,86,87]. Similar 3C chromatin interaction analysis conducted in the presence of other activated SHRs showed that hormonal activation of ER or GR also enhances pre-existing chromatin interactions instead of creating new ones [14,88,89,90]. This loop extrusion process necessary for gene transcription initiation does not seem to precede nascent mRNA production but rather occurs simultaneously with mRNA synthesis, confirming previous studies suggesting a correlation between high RNA production and elevated chromatin contact events [80,91]. However, most genes are not solely reliant on a single CRE to initiate or suppress gene transcription. Instead, they engage multiple CREs with varying regulatory efficacy to fine-tune the level of gene transcription, highlighting the uneven contribution of CREs on gene transcription, where some exert a more pronounced regulatory influence due to higher chromatin contact frequency [92]. Perturbation studies focusing on both low- and high-contact CREs of well-characterized AR target genes reveal that the inactivation of a single high-contact CRE leads to a substantial downregulation, whereas the inactivation of one or more low-contact CREs had a minimal effect [80,91]. 

Chromatin structuring complexes are known to play a facilitating role in promoting CRE-PCI interactions, but their involvement in SHR biology, in general, and AR biology, in particular, remains partially unexplored. Nevertheless, several SHRs and components of the cohesin have recently been reported to be involved. For example, Hager and coworkers demonstrated that GR interacts with the cohesin loading factor NIPBL, promoting long-range gene regulation via loop extrusion by stabilizing NIPBL at GR enhancers [93]. Additionally, PAXIP1, a known AR-interacting protein initially identified as part of the histone modifier complex MT2D/C, was reported to interact with the cohesin complex via STAG2 to regulate SHR-mediated gene expression [94]. Loss of PAXIP1 resulted in an affected frequency of promotor-enhancer interactions and decreased cohesin stability at SHR-bound sites [14,95]. The importance of cohesin complexes in SHR biology was further supported by the observations in cell lines unresponsive to steroid hormonal stimulation due to cohesin mutations [93]. TADs harboring interacting promoters and enhancers in PCa cells correlated with local enrichment of CTCF, cohesin proteins, and AR/FOXA1 complexes to regulate gene transcription, while TAD rearrangements in castration-resistant prostate cancers (CRPC) affected the frequency of CRE-PCI interactions, resulting in altered transcriptional programming [84]. Furthermore, AR upregulation showed a strong correlation with increased expression of bromodomain proteins that are held responsible for inducing chromatin decompaction in CRPC, primarily by BRD2 and BRD4 complexes [96]. Interestingly, BRD4 complexes were also recently reported to interact directly with the cohesin loading factor NIPBL. Removal of BRD4 resulted in reduced NIPBL binding to chromatin and affected chromatin folding facilitated by cohesin, suggesting a potential role for the AR-BRD4 axis in AR-driven gene regulation [97]. Interestingly, another level of AR gene transcription regulation has been reported, in which a specific subgroup of long non-coding RNAs (lncRNA), known as enhancer RNAs (eRNA), are involved in promoting CRE-PIC interactions [98]. For instance, it has been shown that eRNA molecules transcribed from an enhancer of the AR-regulated *KLK3* locus induce *KLK2* gene transcription by facilitating the physical interaction between the *KLK3* enhancer and *KLK2* promotor, aided by MED1 proteins [99]. Furthermore, other lncRNAs are reported to play an important regulatory function in AR biology. For instance, *ARLNC1* has been shown to microscopically colocalize with AR mRNA transcripts in order to stabilize the mRNA molecules [100]. Other lncRNAs (e.g., *PRKAG2-AS1*, *NXTAR*) have been identified over the past few years to regulate specific AR splice variants [101,102], whereas others are reported to form direct interactions with chromatin-bound ARs to regulate AR-mediated gene transcription (e.g., *PGCEM1*, *SOCS2-AS1*) [103,104]. However, it remains elusive whether these AR-interacting lncRNAs, along with other members of the AR interactome, are physically present in AR foci.

Furthermore, the complexity of AR-driven gene regulation is underscored by the widespread distribution of AR-binding sites throughout the genome. ChIP-seq experiments for ARs reveal that ARs bind to thousands of ARBS throughout the genome, whereas the number of genes that are differentially expressed upon hormone activation is in the range of several hundred [105,106]. Since only a minority of AR-binding sites are located at promoter regions, it suggests that ARs primarily regulate gene transcription from several distal CREs [87,107]. Intriguingly, antibody-based chromosome conformation capturing (ChIA-PET) of AR-specific genomic loci showed that over 50% of AR-bound genomic locations identified by ChIP-seq are interacting together to form loops, stabilizing the spatial configuration of several distal AR-binding sites [87]. Although establishing the functional link between these chromatin loops in the 3D genome organization and the location and quantity of AR foci remains challenging, the relative immobility of AR foci and the fact that AR-bound chromatin is stabilized in chromatin loop conformations suggest a mechanistic relationship between these anchor sites and AR foci. 

## 5. Androgen Receptor Foci: Phase-Separated Gene Transcription Regulatory Hubs?

**Figure 4:**
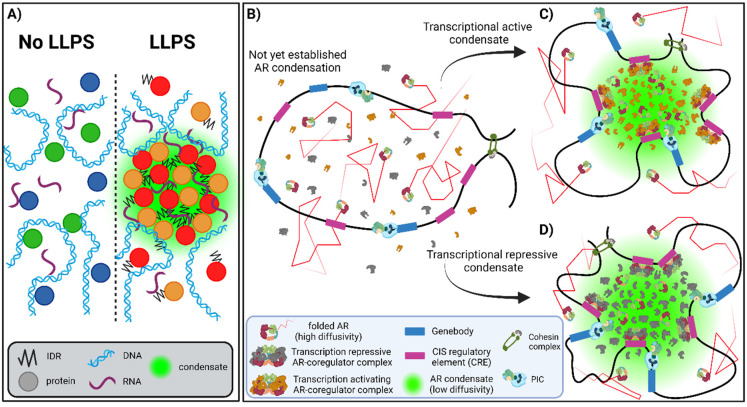
AR condensation in live cells. (**A**) schematic illustration showing protein condensation process known as LLPS. Proteins consisting intrinsically disorder regions (IDR), such as AR, are able to form membrane-less compartments in the nuclear environment together with cofactors and other biomolecules such as DNA and RNAs. (**B**) Mobile, highly diffusive ARs in N/C conformation are not yet nucleated on the DNA upon testosterone stimulation. (**C**,**D**) Shortly afterwards, AR condensation takes place, forming either transcriptionally active condensates (**C**) (containing coactivators such as HATs) or transcriptionally repressive condensates (**D**) (consisting corepressors such as HDACs) which both consists of confined molecules with a low diffusivity as consequence of direct DNA-binding and weak protein-protein interactions.

Besides regulating TF accessibility to CREs, the cooperative binding of ARs to the DNA serves the purpose of creating an interaction platform for TFs that do not always bind directly to DNA, such as MED1, BRD4, and SRC family members [59,107,108,109,110,111]. Interestingly, recent structural analysis has revealed that most of these cofactors, similar to SHRs, including AR, possess intrinsically disordered regions (IDRs) rich in charged amino acids such as prolines, lysines, and arginines, which are believed to facilitate weak protein-protein interactions that establish compartmentalization without the need for membranes [25,112,113,114]. This process is also known as liquid–liquid phase separation (LLPS) [115]. In the chromatin environment, LLPS might be more favorable for compartmentalization compared to the formation of membrane-bounded structures. Many studies on nuclear dynamics of TFs, including SHRs, suggest that DNA-binding is required to generate these membrane-less compartments through LLPS [116], whereas weak protein-protein interactions allow for a more efficient AR-DNA association, thereby facilitating the formation of structures known as microscopically discernible condensates or foci [112,117]. LLPS in the nuclear compartment, characterized as a dynamic and rapidly reversible process, involves intracellular molecules such as water, proteins, and nucleic acids (Figure 4A) [112,118,119]. As certain molecules exceed a concentration and solubility threshold, it becomes thermodynamically beneficial to aggregate into biomolecular condensates that persist until the free energy of the medium changes, for instance, through temperature or pH fluctuations [120,121]. This balance of energy keeps the condensates stable as the environment reaches a thermodynamic equilibrium in which the forces driving molecules to stay together are in harmony with the surrounding conditions [122]. Once established, biomolecular condensates play pivotal roles by increasing the local availability of molecules or disrupting the stability of molecular complexes, thereby influencing the transmission of signals. For example, they can prevent HDACs from entering transcriptionally activating condensates, thus avoiding the suppression of transcriptional active processes [123,124]. Additionally, they may also function as reservoirs of specific molecules required for nearby processes [34,125], thereby allowing rapid regulation of biochemical processes they facilitate to maintain cellular homeostasis [3,29,126].

Notably, MED1 has been demonstrated to colocalize with SHRs such as GR, ER, and AR, displaying morphological characteristics similar to those of other phase-separated condensates, such as nuclear speckles and DNA-repair factors [8,25]. The Mediator complex plays a crucial role during the gene transcription initiation process orchestrated by ARs [17,127] by forming direct interactions with the PIC at promoter regions, where it binds TFIIH to phosphorylate serine 5 of RNA Pol II in order to initiate gene transcription [108,127]. Phase separation could play a pivotal role in this process by rapidly facilitating and increasing the availability of proteins via the IDR of coregulators, such as MED1. The disruption of AR LLPS by LLPS-disrupting substances such as 1,6-hexanediol was recently reported to strongly reduce AR foci, while MED1 presence at ARBS was also significantly reduced [25]. However, it could be argued that 1,6-hexanediol disrupts more than LLPS alone, and more subtle approaches might allow for a more detailed investigation of the LLPS process. Furthermore, the role of LLPS during transcription initiation by the AR-MED1 axis has been illustrated by biochemical assays, such as in vitro droplet assays, providing support for MED1-mediated phase separation via its IDR in conjunction with the full-length AR [25]. A LXXLL motif within MED1 suggests a potential role for the AR’s cofactor-binding groove of the LBD, along with the NTD, in promoting phase separation [17,128]. Moreover, peptide domain fusions of the AR-NTD, AR-DBD, or AR-LBD with the OptoDroplet system to modulate LLPS upon blue light illumination showed that only Opto-AR-NTD possesses intrinsic LLPS-capacity by forming droplets inside cells, whereas Opto-AR-DBD and AR-LBD did not, thereby confirming the essential role of the AR’s NTD as an IDR in LLPS [8]. The facilitating role of the NTD in LLPS has also been observed for GR through SPT experiments, which showed a strongly reduced immobilization of NTD-truncated GR proteins [113]. However, it is worth noting that the structural integrity of the GR-NTD, in contrast to the LBD, is not essential for the formation of GR foci [32]. While the NTD of AR, acting as an IDR, and the DBD, responsible for stable DNA interactions at ARBS, are proposed to play a predominant role in promoting AR phase separation, it is important to emphasize that the overall structural arrangement of ARs remains equally vital for the LLPS of AR foci, as demonstrated in recent research concerning the LLPS capabilities of the DBD [129]. In one study, it was reported that the AR DBD can weakly bind RNA molecules to generate RNA-dependent phase-separated AR protein droplets, as demonstrated through droplet assays. Importantly, this might, in turn, facilitate RNA-dependent LLPS of ARs at gene transcription hotspots by forming interactions with RNA molecules such as eRNAs, which are known to play a role in AR-driven chromatin looping required for promotor-enhancer interactions [99]. However, this phenomenon significantly depends on the relative local concentrations of RNA and DNA since higher DNA concentrations were found to reduce LLPS between ARs and RNA [129]. 

In its entirety, this leads to a hypothetical model in which ARs undergo LLPS during foci formation via a series of distinct interaction events (Figure 4B–D). Initially, multiple AR molecules establish direct interactions with ARBS, followed by interactions with specific coregulators at these DNA anchor sites via their transactivation units of the NTD and via the cofactor-binding groove of the LBD, allowing further compartmentalization driven by phase separation. Interactions with gene transcription-activating coregulators were often reported to be present in AR foci, whereas the dynamic mechanism of transcriptional repressors in AR signaling is not fully understood. Nevertheless, HDAC-recruiting corepressors, such as NCoR and SMRT, were also reported to colocalize with AR foci, indicating that AR foci are not solely associated with gene transcription activation, suggesting that a diverse array of protein interactions contributes to the protein condensation process of either gene transcription-activating or gene transcription-repressive condensates (Figure 4C,D) [36,37,38]. These condensates consist of tens of nucleated ARs from several distant ARBS that are localized in close proximity. Simultaneously, the pre-established three-dimensional chromatin organization facilitates interactions between distant regions (e.g., promoter-enhancer interactions), regulated by chromosome structuring proteins such as the cohesin complex, hypothetically in compartmentalized structures known as AR foci.

## 6. Therapeutic Targeting of the AR’s LLPS Capacity in Disease

Liquid–liquid phase separation in healthy cellular conditions plays a vital regulatory role in many cellular processes by, for example, facilitating gene transcription, chromosome folding, and cellular signaling [130]. However, proteins that are able to undergo LLPS under physiological conditions have also often been found to play a pathological role in, e.g., amyloid-β aggregation in Alzheimer’s disease [131] and oncogenic protein condensates, as described in various types of cancer [130]. 

With the emergence of the LLPS theory and the growing scientific interest in studying LLPS in the context of disease, it has become evident that alternative therapies can be developed by targeting the phase separation capacity of oncogenic driver proteins, including ARs [8]. General, pan-LLPS-disrupting compounds, such as the aliphatic alcohol 1,6-hexanediol, have been shown to dissolve LLPS-driven protein condensates. However, chemicals like 1,6-hexanediol do not fully disrupt all possible interactions (e.g., electrostatic interactions) occurring between IDR-containing proteins. As a result, certain structures may remain unaffected by these compounds, making it difficult to predict the efficacy of the LLPS disruption since it can differ based on protein composition and folding [132]. Furthermore, the use of these general pan-LLPS-disrupting chemicals requires caution, as they also induce drastic morphological alterations of basal organelles, such as membranes, making it impossible to specifically target only one oncogenic driver, such as ARs [133]. This non-specific inhibition of LLPS limits the therapeutic potential of these pan-LLPS compounds because of the general importance of LLPS in cellular processes. 

Instead, protein-specific targeting aimed solely at disrupting the intrinsic LLPS capacity of specific oncogenic proteins is considered a more feasible approach to minimize off-target effects. Compounds that reduce the intrinsic LLPS capacity of AR, such as ET516 and EPI-001 (and it derivates EPI-506/EPI-7386), have also been shown to significantly diminish the transactivation activity of both full-length ARs and their splice variants, highlighting the relationship between protein condensation and gene transcription [8,134,135,136,137]. Furthermore, the potent derivate of EPI-001, EPI-7386 (also known as Masofaniten) is currently in clinical trials for mCRPC patients. These trials have shown encouraging results regarding safety and anti-tumor efficacy, particularly when used in combination with conventional therapeutics like Enzalutamide and Abiraterone [136,137].

## 7. Conclusions 

In recent years, investigations into the role of phase separation in nuclear processes involving SHRs have provided a better understanding of the mechanisms by which they exert their biological functions. Experiments focusing on different receptor domains have revealed a strong correlation between the presence of AR foci and the stable DNA-binding capacity of ARs. Consequently, these observations, along with the presence of nascent RNA and AR coregulators with intrinsically disordered regions (IDRs) required for gene transcription regulation in these foci and the fact that targeting the IDRs of AR/coregulators leads to a reduction in AR foci and transcription activity, have led to hypotheses regarding AR foci as phase-separated protein assemblies facilitating gene transcription. Such hypotheses have, in turn, generated new avenues of research questions with potential implications for modulating LLPS as a means to achieve therapeutic outcomes.

## 8. Knowledge Gaps

Despite decades of multidisciplinary research having revealed the highly dynamic nature of many nuclear processes, the mechanisms driving the spatio-temporal organization of AR-mediated gene transcription in sub-nuclear clusters corresponding to AR foci remain only partially understood. The role of LLPS in forming AR-mediated sub-nuclear transcription foci has become a widely accepted model, supported by the identification of intrinsically disordered regions (IDRs) in several AR transcription initiation complex components and their sub-nuclear distribution in foci. However, many aspects of AR LLPS remain unclear. For example, do the spatio-temporal characteristics of AR condensates differ between ARs expressed in different AR-positive organs (e.g., prostate, breast, liver, bone, muscle, and brain)? And are the known LLPS factors able to initiate these clusters in a regulated fashion, or is their formation largely stochastic? Are ligand-activated ARs and their cofactors clustered within pre-established LLPS clusters, or do they contribute to the formation of de novo clusters? Additionally, how dynamically are ARs and their cofactors exchanged between LLPS clusters and the surrounding nucleoplasm? Although developments in the field of proteomics and sequencing have allowed researchers to characterize the AR interactome in single-cell analyses, the exact protein and RNA (e.g., lncRNAs) composition of individual AR foci and its effects on transcription regulation remain unanswered. For example, does the molecular composition vary between AR foci within a cell nucleus or between cells? If so, what determines the differences in protein composition among AR foci?

It is evident that stable DNA binding of ARs is essential for the formation of AR foci. Visualization of transcriptionally active regions through fluorescent labeling has revealed that AR foci colocalize with sites of active gene transcription. However, the lack of stable DNA-binding in truncated but transcriptionally active ARs lacking the LBD (e.g., AR-v7) shows that stable DNA-binding and the consequential distribution of wild-type ARs in LLPS foci are not strictly required for their transcriptional activity. Therefore, it can be hypothesized that LLPS of AR molecules serves the purpose of enhancing the efficiency of AR-regulated gene transcription by increasing local protein concentrations close to CREs and PICs. Chromatin conformation capturing data indicates that several CREs and AR-regulated genes form loops organized in TADs, whereas labeling of AR-regulated genes in living cells revealed that these genes are in very close proximity to AR foci. However, the level of specificity within the foci is currently unclear: are only genes mediated by individual transcription factors, like AR, colocalized within individual foci, or are foci a more general cluster of transcriptionally regulated genes? And how strictly regulated is the clustering of transcriptionally activated/repressed genes in foci? Moreover, there are still no data that show both CREs and genes are simultaneously organized and regulated spatio-temporally in specific regions of the nucleus that overlap with an AR focus. Answering these long-standing questions will shed light on the causal relationship between the AR’s LLPS capacity and its function as a transcription factor in the context of gene regulation in PCa.

Understanding the mechanisms and processes underlying the spatio-temporal regulation of AR-orchestrated genes in the context of AR nuclear condensates could eventually lead to the development of a new generation of compounds to affect the LLPS in disease as a method to target dysregulated AR transcriptional programming. Several AR LLPS-reducing compounds targeting the NTD have already been shown to significantly reduce the AR’s transcriptional activity. However, the precise mechanism by which these compounds minimize LLPS remains unclear at the molecular level. A proteomic analysis of cells treated with the NTD inhibitor EPI-001 revealed a significant reduction in AR-protein interactions, including MED1, raising the question if these interactions are lost because of LLPS inhibition or vice versa [51]. In other words, do they reduce LLPS simply by inhibiting required cofactor interactions to the NTD, or do they directly impair the intrinsic LLPS capacity of the protein itself, which results in reduced AR condensates? Moreover, even though the NTD of ARs shares less than 15% sequence homology with other SHRs, it cannot be excluded that these compounds lack off-target effects. Thus, further research remains necessary to investigate the specificity of these compounds on the cellular proteome before they can be used as a therapeutic. Eventually, they may be used with AR LBD antagonists (e.g., Enzalutamide) in a synergistic manner, offering new potential therapeutic strategies for patients with castration-resistant PCa.

## Data Availability

No new data were created or analyzed in this study. Data sharing is not applicable to this article.
